# Climate change and allergies

**DOI:** 10.1007/s40629-022-00212-x

**Published:** 2022-06-08

**Authors:** Daria Luschkova, Claudia Traidl-Hoffmann, Alika Ludwig

**Affiliations:** 1 Environmental Medicine, Faculty of Medicine, University of Augsburg, Augsburg, Germany; 2 University Outpatient Clinic of Environmental Medicine, Augsburg University Hospital, Augsburg, Germany; 3 Institute of Environmental Medicine, Helmholtz Zentrum München, Munich, Germany; 4 CK-CARE, Christine Kühne—Center for Allergy Research and Education, Davos Wolfgang, Switzerland

**Keywords:** Heat wave, Air pollution, Pollen allergenicity, Ragweed, Thunderstorm asthma, COVID-19

## Abstract

The climate crisis poses a major challenge to human health as well as the healthcare system and threatens to jeopardize the medical progress made in recent decades. However, addressing climate change may also be the greatest opportunity for global health in the 21st century. The climate crisis and its consequences, such as rising temperatures, forest fires, floods, droughts, and changes in the quality and quantity of food and water, directly and indirectly affect human physical and mental health. More intense and frequent heat waves and declining air quality have been shown to increase all-cause mortality, especially among the most vulnerable. Climate warming alters existing ecosystems and favors biological invasions by species that better tolerate heat and drought. Pathogen profiles are changing, and the transmission and spread of vector-borne diseases are increasing. The spread of neophytes in Europe, such as ragweed, is creating new pollen sources that increase allergen exposure for allergy sufferers. In addition, the overall milder weather, especially in combination with air pollution and increased CO_2_ levels, is changing the production and allergenicity of pollen. The phenomenon of thunderstorm asthma is also occurring more frequently. In view of the increasing prevalence of allergic diseases due to climate change, early causal immunomodulatory therapy is therefore all the more important. During a climate consultation, patients can receive individual advice on climate adaptation and resilience and the benefits of CO_2_ reduction—for their own and the planet’s health. Almost 5% of all greenhouse gas emissions in Europe come from the healthcare sector. It thus has a central responsibility for a climate-neutral and sustainable transformation.

In 2015, the Lancet Commission on Health and Climate Change noted that “that tackling climate change could be the greatest global health opportunity of the 21st century”, while warning that climate change “threatens to undermine the last half century of gains in development and global health” [1]. UN Secretary-General Antonio Guterres noted in 2020 that human activities “are at the root of our descent toward chaos. But that also means human action can help to solve it” [2].

## Temperature and pollutant exposure and the effect on health

The United Nations Intergovernmental Panel on Climate Change (IPCC) states in its 2021 Assessment Report that the increasing frequency and intensity of heat extremes, marine heat waves, heavy precipitation, agricultural and environmental droughts, and the rate of severe tropical cyclones are directly related to global warming [3].

July 2021 was the hottest month globally since records began 142 years ago [4]. Exposure to heat, in addition to mental as well as physical performance decline, also leads to increased all-cause mortality [5, 6]. The elderly, young children, and the chronically ill are particularly vulnerable, especially those with pulmonary or cardiac pre-existing conditions, renal insufficiency, dementia, or diabetes mellitus [7]. In addition, socioecological factors, such as living in densely built-up urban areas, play a role. In Germany, 75% of the population lives in cities. In these, there is a heat island effect, synergistically reinforced by increased ozone and particulate matter levels.

Temperature increases favor wildfires, which, in addition to causing immediate deaths, lead to posttraumatic stress disorder and increased cardiovascular and respiratory mortality due to the air pollutants released [6]. In addition to forest fires, anthropogenic emissions are responsible for declining air quality. According to the WHO, the most important substances harmful to health are ozone, nitrogen dioxide, sulfur dioxide and particulate matter [8]. Other air pollutants include gases such as benzene, toluene, xylene, liquid aerosols (perchloroethylene, methylene chloride), inhalable particulate-bound pollutants such as polycyclic aromatic hydrocarbons (PAHs), cadmium, chromium, lead, and mercury. Emissions have long atmospheric residence times and can travel long distances across continents and oceans. Cumulative damage, oxidative stress, proinflammatory and inflammatory responses, and epigenetic changes are possible consequences [6]. Expanding research in this area is essential [9].

Due to climate change, mental illnesses such as depression or posttraumatic stress disorder are on the rise [6], but also cardiovascular and metabolic diseases, e.g., diabetes mellitus [10] allergies and especially vector-borne infections [6, 11]. Factors that accelerate climate change, such as the destruction of natural habitats and of animal and plant species, also increase the risk of pandemics, such as the current SARS-CoV‑2 (severe acute respiratory syndrome coronavirus type 2) pandemic [12]. Flooding can lead to, among other things, increased exposure to molds and dust mites, and thus secondary to asthma and allergic rhinitis [4].

## Increase in allergic diseases

In recent decades, allergic diseases have spread epidemically. More than 128 million Europeans are affected. Especially in the younger European population, allergies are widespread with more than 30%. Allergies are caused or triggered by environmental factors and are exacerbated in prevalence, phenotypic expression, and severity by climate change and air pollution [9]. In addition to loss of quality of life, they lead to enormous damage of a socioeconomic nature. Air pollutants such as ozone, nitrogen oxides (NOx), and ultrafine particles fuel inflammatory processes in the mucous membranes of the upper and lower respiratory tract. In addition, air pollutants and aeroallergens interact with each other [9]. Urbanization also contributes to the development and worsening of atopic dermatitis through air pollutants and the decrease in biodiversity [13].

## Changes in aeroallergen exposure

Climate change and air pollution have an effect on the release and dispersion of airborne pollen and thereby on allergic diseases (Table 1).

**Table 1:** Summary of climate change impacts on pollen

Shift of vegetation zones
Changes in onset and duration of the pollen season
Increase of airborne pollen concentration
Immigration and spread of neophytes (e.g., *Ambrosia artemisiifolia*)
Increase of pollen allergenicity: changes in allergenic proteins and adjuvant substances in pollen (e.g. pollen-associated lipid mediators (PALMs), lipopolysaccharide (LPS))
Thunderstorm asthma: increased exposure to small allergen fragments during thunderstorms

→ Effect on time span and severity of symptoms in patients with allergies

## Changes in pollen season and airborne pollen concentration

Phenological characteristics of plants such as flowering and pollen production are very sensitive to environmental changes. Global warming, especially in combination with increased CO_2_ (a natural fertilization effect), changes the intensity and timing of flowering, causes a shift and an extension of the growing season and an increase in plant biomass. This results in a seasonally earlier pollen onset, the extension of the pollen season, the appearance of new allergenic plants and their pollen in Europe (e.g., *Ambrosia artemisiifolia*, olive trees, Parietaria, cypress) and, in combination with air pollutants, an increase in pollen allergenicity and airborne pollen concentration [6, 9]. This affects both the time period and the symptom severity of the allergy sufferers’ symptoms. The greater exposure to allergens also increases the possibility of new sensitization.

For example, the first hazel bushes of the reference year 2016 already bloomed in western Germany at the beginning of December. Thus, the beginning of the meteorological winter coincided with the beginning of the phenological early spring there [14]. Due to cross-reactions to hazel and alder pollen, persons allergic to birch pollen can also show symptoms as early as December. It is important to predict the local pollen load (pollen count, long-distance transport) as accurately as possible with the help of pollen monitoring, so that those affected can adjust to the allergen exposure in good time. It should be noted that changes in the pollen spectrum and pollen emission are not only dependent on climatic factors, but are also influenced, for example, by agricultural activities and changes in land use and microclimate as a result of increasing urbanization [15].

## New pollen sources in Europe

The general conditions—increasingly warmer and drier summers and milder and more rainy winters—are leading to changes in existing ecosystems, the species spectrum and shifts in vegetation zones. The abundance of heat-loving and drought-tolerant species increases. In parallel, cold-adapted species are displaced to more northerly regions and higher altitudes. Low-allergen refuges for people with allergies, such as the Alps, are lost. In addition, an invasion of alien species is favored.

**Fig. 1:**
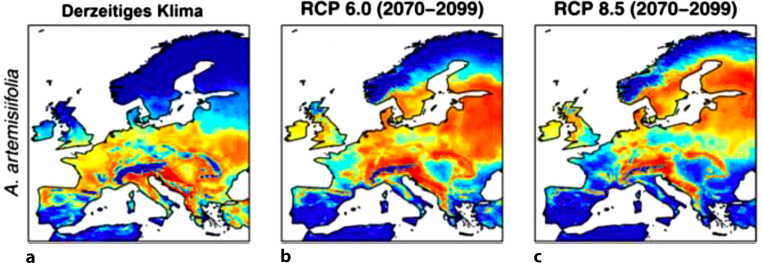
Habitat suitability in Europe for *Ambrosia artemisiifolia *under current climate conditions and the future IPCC climate scenarios RCP 6.0 and RCP 8.5 for the years 2070–2099. Translation figure legend: *Derzeitiges Klima* current climate. (Modified from Rasmussen et al. [17], Creative Commons CC-BY 4.0)

Germany’s invasive species number in the thousands. Previously alien plants, such as glasswort, olive trees, and cypress, are becoming native to Germany, providing new sources of pollen [9]. From an allergological point of view, *Ambrosia artemisiifolia* (ragweed) in particular deserves special attention as a neophyte from North America. The climatic changes of the last decades favor the naturalization and spread of the alien plant. Since the 1980s, a rapid expansion in Central Europe has been recorded [15]. The plant spreads mainly in ruderal areas, fallow land and along traffic routes [15]. The allergenic potential of this plant is considerable. The inflorescences produce an enormous amount of pollen, about 1 billion pollen, which are also smaller than grass pollen and can spread over longer distances. The late main flowering period (August–September) prolongs the symptoms of specifically sensitized individuals into the fall. Even low concentrations of 5–10 pollen grains per cubic meter of air are sufficient to trigger allergic and especially asthmatic symptoms [11]. The prevalence of sensitization to *Ambrosia artemisiifolia *is rapidly increasing (currently over 8% of German adults) [16]. In addition, cross-reactivity to mugwort is problematic. In this context, new sensitization to ragweed pollen is often not necessary to cause allergic symptoms [9]. A complete eradication of the neophyte is no longer considered realistic (Fig. 1).

## Increase in airborne pollen allergenicity

Air pollutants and climate scenarios such as high CO_2_ or drought act as additional stressors on plants. They often respond by altering allergen-encoding transcripts, protein and metabolite profiles and increase the allergenicity of their pollen. Especially in urban microclimates or along high-traffic roads, polluted air increases pollen concentrations and plant allergenicity [18]. In studies, for example, birch pollen in regions with a high atmospheric ozone content was found to have a stronger allergenicity. In the pollen samples, not only the amount of the main allergen *Bet v 1*, but also adjuvant substances (such as pollen-associated lipid mediators [PALMs], lipopolysaccharide (LPS), adenosine) were modulated [19, 20]. These have both proinflammatory and immunomodulatory effects and may promote or exacerbate allergy [18]. Both climate chamber and field experiments show increased allergenicity of ragweed pollen at higher concentrations of air pollutants. An increase in CO_2_ levels and drought stress result in significantly increased ragweed pollen production. In addition, the ragweed major allergen *Amb a 1 is *increasingly produced under such conditions [21]. Higher NO_2_ concentrations can cause the formation of new allergens in pollen [22]. Pollen can also attach to particulate matter and diesel exhaust particles, leading to a stronger allergic effect when inhaled in this combination. In addition to pollen, allergenicity may also increase in fungal spores as a result of climate change.

## Thunderstorm asthma

**Fig. 2:**
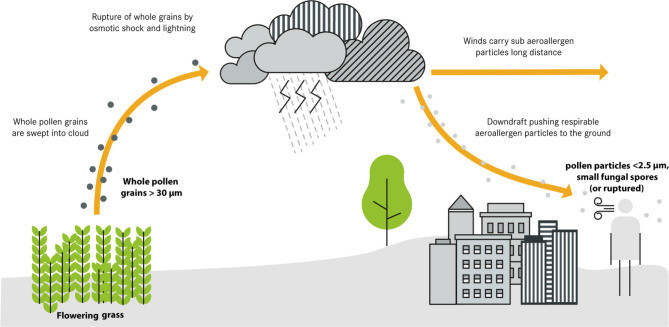
In “thunderstorm asthma,” aeroallergens are swept into the cloud systems, ruptured through osmotic shock and lightning activity, releasing a variety of respirable allergenic particles. Strong winds can transport these particles over long distances. Downdrafts can concentrate the particles near the ground, leading to a large increase in allergen content in the ambient air. (Modified from Chatelier et al. [23], Creative Commons CC-BY 4.0)

The phenomenon of “thunderstorm-induced asthma” or “thunderstorm asthma” refers to the clustered occurrence of partially severe asthma attacks in the temporal and spatial environment of thunderstorms. In severe thunderstorms, whose frequency and intensity will increase in the course of climate change, and at the same time high pollen load, asthma exacerbations or severe allergic rhinitis symptoms may occur. The presumed pathomechanism is that pollen (especially from grasses) and fungal spores (Alternaria and Cladosporium) are increasingly stirred up in the run-up to a thunderstorm, swell osmotically due to the electrostatic charge and atmospheric humidity, and rupture. Cytoplasmic components present in pollen grains are released into the ambient air. This results in smaller, more respirable pollen fragments penetrating deeper into the bronchial system than usual and can lead to acute bronchospasm (Fig. 2; [23, 24]). The exposure situation for persons sensitized to the allergen worsens massively. Persons with allergic rhinitis may also suddenly suffer severe bronchial obstruction and asthma attacks [9]. A significant risk exists especially in patients with inadequately treated asthma.

One of the largest thunderstorm-related asthma events occurred in Melbourne, Australia. In this event, the number of emergency admissions due to respiratory problems after a storm on November 21, 2016, increased by 672% within a few hours (3365 cases more than expected). This resulted in numerous treatments in intensive care units and a total of ten deaths [23]. The phenomenon of “thunderstorm asthma” is also becoming more common in Europe [25].

## Pollen and SARS-CoV-2 viruses

Exposure to pollen weakens the immune response to certain rhinoviruses by reducing the interferon response, regardless of the presence of allergy. This is also true for SARS-CoV‑2 (severe acute respiratory coronavirus disease 2) viruses, as shown in a global epidemiological study [26]. Mouth-to-nose protection may not only reduce the transmission of SARS-CoV‑2 viruses, but also the uptake of pollen. The authors therefore recommend wearing FFP2 masks in pandemic times even outdoors, especially when pollen counts are high [11, 26].

## Increased prevalence of the oak processionary moth

Oaks can harbor the caterpillars of the oak processionary moth (Thaumetopoea processionea), which are significant from an allergological point of view [11, 27]. This moth has been on the rise in Europe for years, also in Germany. The frequency of occurrence can only be estimated. Burning hairs of the caterpillars, invisible to the human eye, which can be carried far through the air, trigger toxic and also IgE-mediated reactions. Symptoms include urticaria and other skin reactions, conjunctivitis, respiratory distress, and even anaphylactic shock. Climate change favors earlier sprouting and growth of oaks. From a health protection point of view, control of the caterpillars or barriers around infested trees near kindergartens, schools or recreational areas are necessary.

## New food allergens

Climate change can, for example, slow food production through droughts or floods to the point of famine [5]. Therefore, insects, for example, are interesting as new food (sources), which, as a protein-rich alternative for animal foods, can in turn slow down climate change [5]. The larvae of the flour beetle (Tenebrio molitor), house crickets (Acheta domesticus), and European migratory grasshoppers (Locusta migratoria) have so far been approved as novel foods in the EU [28]. Anaphylactic reactions to insects have been observed, particularly as a result of cross-reactions, for example, to the heat- and digestion-stable pan-allergens tropomyosin and arginine kinase, which are also present in other arthropods, occuring in people allergic to dust mites, cockroaches and crustaceans. However, (allergenic) algae, mammals, plants and fungi could also cross-react with insects [29].

## Conclusion for the healthcare sector

Numerous negative impacts of the climate crisis on health have been recognized. However, for Germany, the Lancet Countdown on Health and Climate Change 2021 noted that in the areas of heat protection, reduction of the health sector’s carbon footprint, and integration of the issue into education, training, and continuing education, “substantial progress in implementing the recommendations has, however, been lacking over the past two years” [30].

In view of the increasing frequency of respiratory allergies due to climate change, early causal immunomodulatory therapy is all the more important. For this purpose, SIT (specific immunotherapy, hyposensitization) is available in subcutaneous or sublingual form and is particularly promising with grass and birch pollen in allergic rhinitis and/or conjunctivitis, but also effective in allergic bronchial asthma. However, there is already a considerable underuse of SIT for affected people in Germany [31]. This has many causes, which should be counteracted (Table 3). There is a great need for research in the field of immunology and allergology. For example, new insights into the mode of action of SIT, its long-term effects as well as suitable biomarkers for the selection of those to be treated and also for the prognosis of therapeutic success could make the care of patients more effective and cost-efficient. The pathomechanisms and molecular processes involved in the development of allergy are still partly unclear [31] (Table 2).

**Table 2:** Research needs for adaptation to climate change

Primary prevention	Funding of basic, applied or clinical research in the field of immunology and allergology: allergen research, research into sensitization pathways, development and chronification of allergic diseases
	Research on protective environmental factors with regard to allergies (biodiversity, traditional lifestyle)
	Exploring the barrier function and microbiome of the skin, gut and respiratory tract
	Significance of psychosocial factors on development and manifestation of allergic diseases
	Changing atmospheric circulation patterns: impact on pollen concentration quantification
	Long-term trends in ragweed allergy in Germany
Secondary prevention	Promote studies on interaction between exposome, environmental risks and allergic diseases; better disseminate their results
	Improvement of molecular diagnostics, development of new allergen therapeutics
	Biomarkers for selection of appropriate patients and prognosis determination for specific immunotherapy (SIT)
	Response to systemic therapies under different environmental factors such as heat, UV radiation, pollen exposure
	Effects of the microbiome on allergic diseases
	Individual pollen monitoring
	Personalized early warning systems (e.g., thunderstorms for pollen allergy sufferers)

**Table 3:** Demands on goverment agencies with regard to allergies

National Allergy Action Plan
Promotion of research in the field of allergology
Allergology as mandatory content in education and training of all health professions and for educating and teaching staff
Creation of separate and independent full professorships for allergology and for environmental medicine
Financial support for information campaigns for the population, e.g., on specific immunotherapy (SIT)
Reimbursement of pharmacotherapy of allergic rhinitis by the German statutory health insurance (SHI)
Preservation of the prescribing and reimbursement eligibility also of rare allergens for SIT
Financial support for the standardization of preparations for SIT and skin testing
Securing funding for development, clinical testing and manufacturing of allergy diagnostics
Reimbursement of patient training courses for neurodermatitis, anaphylaxis and asthma by the SHI system

Prescribing preventive medical examinations or occupational entry counseling for occupations with special hazards [31, 37]

Legislation enshrining health-based heat protection is a prerequisite to prioritizing heat action plans [30]. These should also include action scenarios for exceptionally extreme and complex situations [32]. Appropriate urban planning, e.g., the expansion of parks, street trees and green roofs, can counteract the heat island effect [11, 32]. It is important to work together from the outset in an interdisciplinary and cross-sectoral way, involving urban planners, architects, transport experts and health professionals, among others [33]. The latter in particular have not been involved in many cases to date. This explains, for example, why birch avenues were planted at Potsdamer Platz in Berlin. Planting should therefore (also) be based on the risk of allergies [11].

Counseling patients on individual climate adaptation and resilience can be provided, for example, in the context of a climate consultation [11].

Curricula on climate change and health, as well as planetary health and allergology, should be included in the mandatory curricula, continuing education and training of all health professionals [32, 34].

Healthcare contributes 5.2% of Germany’s greenhouse gas emissions. The WHO initiative “Healthy Hospitals, Healthy Planet, Healthy People” [35] distinguishes seven areas for climate protection in hospitals: energy efficiency, green building design, alternative energy generation, transportation (staff and patient travel), food, waste, and water. Climate protection measures can also be implemented in medical practices, medical associations, at medical conventions, etc. To date, there are no legislative proposals on this at the state, federal or European level [32]. The 125th German Medical Congress in 2021 called for climate neutrality in the German healthcare system by 2030, citing the initiation of the necessary legal framework, the appointment of climate officers and the adoption of climate protection plans as prerequisites. Some initiatives already exist in Germany, such as the “Energy-Saving Hospital” seal of approval, the KLIKgreen project, which trains specialists in hospitals to become climate managers, and the Green Hospital Initiative Bavaria. Important factors are the provision of funding by the federal and state governments, identification and dismantling of legal barriers, and progress monitoring through balancing and reporting of greenhouse gas emissions in the participating facilities [32].

In terms of “planetary health” (Planetary Health), Earth is a medical emergency [36]. It is up to us readers of *Allergo Journal* to stand up, educate, raise awareness, and demand just solutions for climate and health policy [4]. In doing so, physicians can take the leap of faith in the general public to be impetus generators and role models. The decisions we make today, including our individual actions or inactions, will impact future generations. We have a responsibility to reduce the impact of climate change on public health.
